# Low-Power Three-Dimensional Graphene-Based Flexible Magnetic Sensor

**DOI:** 10.3390/polym18040477

**Published:** 2026-02-13

**Authors:** Shiliang Zhao, Yao Wang

**Affiliations:** State Key Laboratory of Submarine Geoscience, School of Automation and Intelligent Sensing, Shanghai Jiao Tong University, Shanghai 200240, China; vector.zhao@sjtu.edu.cn

**Keywords:** graphene, magnetic impedance, magnetoresistance, 3D magnetometer, low power

## Abstract

Flexible magnetic sensors have become a hot research topic due to their non-contact human–machine interaction capabilities in areas such as motion recognition and posture detection of intelligent robots, virtual reality (VR) space reconstruction, and the Internet of Things. This study proposes a flexible, low-power three-dimensional (3D) magneto-impedance (MI) sensor based on a planar FeSiB/PI/graphene microcoil/PI/FeSiB heterostructure. Through the magneto-impedance effect of soft magnetic materials and the magnetoresistance effect of graphene under the synergistic modulation of weak current excitation, this sensor can decouple the magnetic field components in the X, Y, and Z directions with a single measurement, thus guaranteeing the real-time detection capability of a 3D magnetic field. Experimental results show that the proposed 3D magnetic sensor possesses the obvious advantages, such as the low power consumption of 76 μW, high resolutions of 31, 36, and 6992 nT/Hz^1/2^ in the X, Y, and Z directions, respectively. Additionally, the magnetic sensor exhibits excellent anti-bending performance and can adapt to complex mechanical deformation environments. These characteristics endow it with great application potential in the field of intelligent wearable devices and provide new ideas for the future flexible electronics technology.

## 1. Introduction

In recent years, with the rapid development of the Internet of Things (IoT) [1,2], wearable devices [3,4,5], and the intelligent robot [6], flexible magnetic sensors have gradually become a research hotspot. These sensors can be integrated into various wearable devices due to the advantages of being lightweight, soft and flexible as well as simple preparation process, which demonstrates the excellent adaptability and practicality [7,8,9,10,11]. Specifically detecting the spatial distribution characteristics of magnetic field with magnetic sensors provide the advantages of non-contact detection [7,8,12] and possible technological support for the diverse human–machine interaction scenarios. Currently, the flexible uniaxial magnetic sensors based on Hall, anisotropic magnetoresistance (AMR, i.e., Py), giant magnetoresistive (GMR, i.e., Py/Cu multilayers), tunnel magnetoresistance (TMR, i.e., MgO-barrier), magnetoimpedance (MI, i.e., Py/Cu/Py) and magnetoelectric (ME i.e., Metglas/Cellulose film) effects can achieve various functions such as positioning and navigation [13,14], health monitoring [15], biometric identification [16], gesture control [11], and tactile feedback [12,17]. Accordingly, they are widely used in multiple fields such as smart wearable devices, medical diagnosis, virtual and augmented reality (VR/AR), and intelligent robots [11].

With the further advancement of intelligent robot motion tracking and virtual/augmented reality technologies, the demand for 3D magnetic field detection is also increasing. Currently, the applications of flexible magnetic sensors based on various effects are mainly limited to in-plane magnetic field detection (as shown in Table 1) and thus cannot meet the requirements of such complex scenarios. Therefore, the detection of a 3D magnetic field is generally achieved by the magnetic fields in the X, Y, and Z directions being respectively sensed by three single-axis sensors [13,18,19,20,21]. This design, nonetheless, suffers from the inevitable introduction of non-orthogonality errors, spatial error and angular deviations, a drawback that demands complex calibration protocols. Meanwhile, since many sensors are in working state, the overall power consumption of this solution is quite high (i.e., usually reaching the mW level), which limits its applicability in low-power consumption scenarios.

What is more, the 3-D placement of sensors in space is prone to generating extra spatial errors during measurements, thereby exacerbating the system’s complexity and raising its power consumption [22,23]. Therefore, the development of a 3D magnetometer with ultra-low power consumption, planar structure, and easy integration into wearable devices has become an urgent need in the research field.

Christian B. [13] proposed a high-density 3D magnetic sensor integrated with a self-assembled micro-origami cubic structure based on the AMR effect. After embedding this sensor into an electronic skin with magnetic hairs, the multi-directional tactile perception can be achieved in real time. However, this design still orthogonally arranges three uniaxial AMR sensors along the X, Y, and Z directions, making it susceptible to the angular errors.

**Table 1:** Technical comparison of flexible magnetic sensors.

Sensor	Material	Geometry	X-D	Sensitive Direction ^1^	Reference
Hall	Graphene (Gra)	2D	1	z	[24]
AMR	Py	3D	3	x, y and z	[13]
GMR	Py/Cu multilayers	2D	1	x or y	[25]
TMR	MgO-barrier	2D	1	x or y	[26]
MI	Py/Cu/Py	2D	1	x or y	[27]
ME	Metglas/Cellulose film	2D	1	x or y	[28]
MI/MR	Metglas (M)/Gra/M	2D	3	x, y and z	This work

^1^ x/y represents the axis direction in the plane of the device, while z is defined as the direction perpendicular to the device plane.

Notably, the graphene is regarded as a highly promising material in the field of magnetic sensors due to its low carrier concentration, excellent electrical conductivity, and single-atomic-layer thickness. Specifically, its minimal power draw trait (i.e., weak skin effect [29]) and magnetoresistance effect, as well as the good flexibility, make it an ideal candidate for flexible magnetic sensors. Zhenxing W. [24] fabricated a flexible Hall sensor based on graphene with a sensitivity of 79 V/AT and excellent anti-bending performance. Subsequently, Huang L. [30] further improved the sensor performance by optimizing the interface between graphene and the flexible substrate, improving the sensitivity to 437 V/AT. Such performances lead in the field of flexible Hall sensors as well as the traditional silicon-based sensors. However, current research on graphene-based flexible magnetic sensors mainly focuses on their detection ability for vertical magnetic fields, which limits the application in 3D magnetic field measurement.

This study proposes a planar low-power 3D magnetic sensor based on a FeSiB/graphene micro-coil/FeSiB heterostructure. The sensor uses a weak alternating current with a direct current bias to excite graphene, which detects the in-plane magnetic field through the impedance change of soft-magnetic thin film, and simultaneously senses the magnetic field perpendicular to the plane via the magnetoresistance effect of graphene. Therefore, the sensor only needs one excitation to complete the measurement of 3-D magnetic field components. The experimental results show that the sensor performs excellently; its resolutions reach 31 nT/Hz^1/2^ (x-axis), 36 nT/Hz^1/2^ (y-axis), and 6992 nT/Hz^1/2^ (z-axis) respectively, and meanwhile it has extremely low power consumption, only 76 μW. In addition, due to its single-excitation design feature, the sensor has the ability to detect the magnetic field in real time. This study provides a solution with both low power consumption and economy for 3D magnetic field detection, and shows broad application prospects. Especially in scenarios with high requirements for energy consumption and flexibility, such as intelligent robots, implantable medical devices, and smart wearable devices, its potential is particularly prominent.

## 2. Materials and Methods

### 2.1. Materials

Ultra-thin graphene paper with a purity higher than 99% (thickness about 50 μm) was provided by Nanjing XFNANO Materials Tech Co., Ltd. (Nanjing, China). This material was made by pressing single-layer graphene. Soft magnetic ribbons (Metglas 2605, FeBSi) were purchased from Foshan Huaxin Microlite Metal Co., Ltd. (Foshan, China). In addition, polyimide (PI) materials (thickness about 7.5 μm) were provided by DuPont (Wilmington, DE, USA). 1,3-Bis-(2,6-diisopropylphenyl) imidazol-2-ylidene (IPr) was purchased from TCI Development Co., Ltd. (Shanghai, China).

### 2.2. Sensor Fabrication

**Figure 1:**
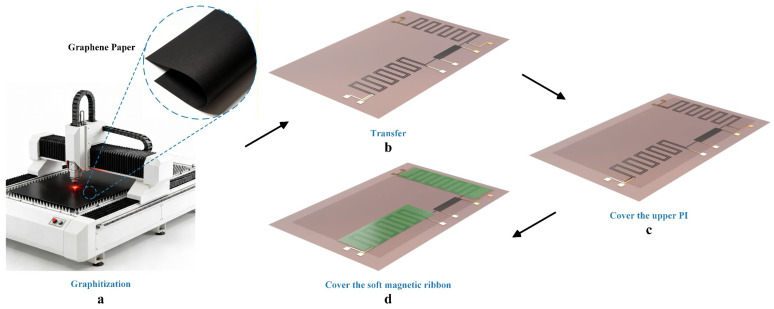
Fabrication process of graphene-based flexible 3D magnetic sensor. (**a**) Graphical processing of graphene paper; (**b**) Transfer the graphically processed graphene to PI substrate; (**c**) Cover and protect with top layer PI; (**d**) Sandwich the graphene zigzag structure with soft magnetic thin ribbons.

The preparation process of the sensor is shown in Figure 1. The preparation process of the sensor is shown in Figure 1. First, the purchased graphene paper is precisely patterned into a zigzag structure (i.e., Figure 1a) using a Silhouette CAMEO 4 cutter (Lindon, UT, USA). After the cutting was completed, the patterned graphene paper was transferred onto a pre-treated polyimide (PI) substrate (i.e., Figure 1b). This substrate had Cr/Au (10 nm/50 nm) electrodes deposited by magnetron sputtering technology, and the electrode surface was pre-treated with a 10 mM IP solution to functionalize the gold electrode surface [31]. After the successful transfer of graphene paper, a top-layer PI film was covered over it to achieve effective insulation between the graphene and the soft magnetic ribbon (i.e., Figure 1c). Finally, the cut soft magnetic ribbon and the meandering structure of graphene paper were clamped and assembled (i.e., epoxy resin) to form the 3D magnetic sensor (i.e., Figure 1d).

## 3. Results

**Figure 2:**
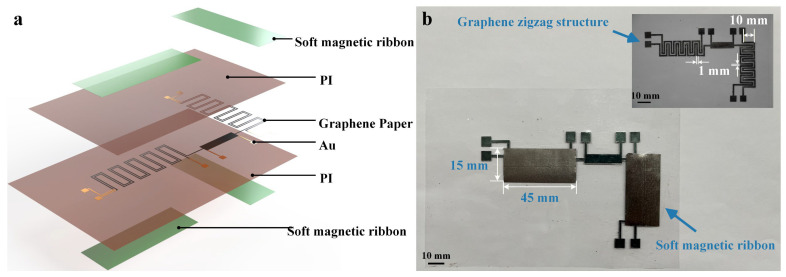
(**a**) Schematic diagram of the flexible 3D magnetic sensor. (**b**) Structural diagram of the fabricated device. The inset in the figure shows the patterned graphene zigzag structure.

Figure 2b depicts the structural layout of the flexible magnetic sensor, where the patterned excitation-sensing graphene coils are sandwiched between the PI films and FeSiB ribbons. Under the action of the out-of-plane magnetic field Hz (z-direction), graphene’s internal carriers are deflected by the Lorentz force, lengthening their transport path and causing the varied magnetoresistance (MR) with *H*_z_ [32]. Meanwhile, when graphene is sandwiched between high-permeability FeSiB ferromagnetic thin films, a distinct magneto-impedance (MI) effect is produced by the graphene micro-coils [33], whose electric output changes with the in-plane magnetic fields *H*_x_ and *H*_y_.

**Figure 3:**
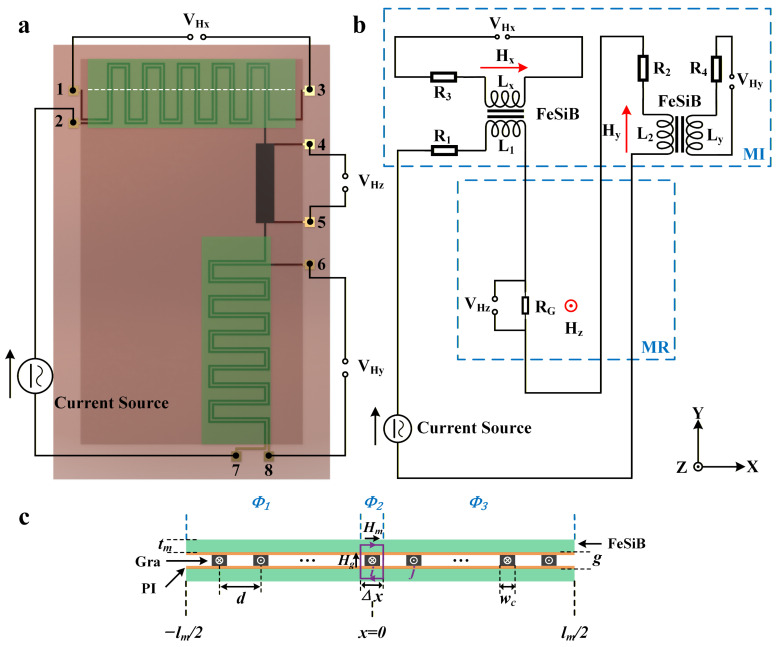
(**a**) Top view of graphene-based 3D magnetic sensor; (**b**) Equivalent circuit diagram of the sensor; and (**c**) The cross-section along the white dotted line in (**a**).

Here, a planar micro-coil structure is fabricated by patterning graphene. This structure consists of an excitation coil and two orthogonal sensing coils based on zigzag-etched graphene strips, which are metallized through eight metal pads (Figure 3a). Specifically, the excitation coil is driven by applying an alternating current to pads 2–7, and the magnetoresistance effect corresponding to the magnetic field in the z-direction can be readout through pads 4 and 5. Meanwhile, by utilizing the inductive coupling between the excitation coil and the orthogonal sensing micro-coils, the magneto-impedance effect responding to the magnetic fields in the x-direction and y-direction can be measured at pads 1 to 3 and pads 6 to 8, respectively. Based on the above design, this single device can achieve the accurate sensing of a 3D magnetic field.

### 3.1. Equivalent Circuit Model

The equivalent circuit model of the 3-D flexible magnetic sensor based on graphene is shown in Figure 3b. Here, R and L represent the resistance and inductance of the graphene microcoil on the soft magnetic film, respectively. The direction of the magnetic field is also marked in the figure: H_x_ and H_y_ represent the in-plane magnetic fields of the device, along the width and length directions of the device, respectively, and H_z_ represents the out-of-plane magnetic field, perpendicular to the device surface.

Under the action of the excitation current I (i.e., including DC bias), according to the theoretical models of MI [33] and MR [32], the impedance of this three-dimensional graphene magnetic sensor can be expressed as:

(1) $$Z_{Hx}=V_{Hx}/I=\omega M_{x}=\omega k\sqrt{L_{1}L_{x}}=\omega L_{x}$$

(2) $$Z_{Hy}=V_{Hy}/I=\omega M_{y}=\omega k\sqrt{L_{2}L_{y}}=\omega L_{y}$$

(3) $$R_{Hz}=V_{Hz}/I_{DC}=R_{G}$$

In the formula, *ω* is the frequency of the excitation current I; *M*_x_ is the mutual inductance between *L*_1_ and *L*_x_, and *M*_y_ is the mutual inductance between *L*_2_ and *L*_y_; k ≈ 1 is the mutual coupling coefficient (for details, see Supplementary Material S1). *L*_1_, *L*_2_, *L*_x_, and *L*_y_ denote the inductance values of the graphene coil, respectively. *R_G_* refers to the magnetoresistance varying as a function of *H*_z_ as follows:

(4) $$\frac{R_{G}\left(H_{z}\right)-R_{G}\left(0\right)}{R_{G}\left(0\right)}=\frac{{∆R}_{G}}{R_{G}\left(0\right)}=\beta \frac{H_{z}^{2}}{3H_{t}}\;(H_{z}\;\leq \;H_{t})$$

(5) $$\frac{R_{G}\left(H_{z}\right)-R_{G}\left(0\right)}{R_{G}\left(0\right)}=\frac{{∆R}_{G}}{R_{G}\left(0\right)}=\beta \left(H_{z}-H_{t}+\frac{H_{t}^{2}}{3H_{z}}\right)\;\left(H_{z}\;\geq \;H_{t}\right)$$

Here, β and H_t_ are both constants. Specifically, the magnetoresistance varies differently depending on the relative magnitude of *H*_z_ and H_t_. When the magnetic field *H*_z_ is smaller than H_t_, the ratio Δ*R_G_*/R_G_(0) shows a positive correlation with $H_{z}^{2}$. For comparison, the ratio Δ*R_G_*/R_G_(0) as a function of H_z_ becomes more complex when the magnetic field H_z_ is larger than H_t_.

The core mechanism of the magneto-impedance effect of this sensor lies in that the in-plane magnetic fields (i.e., H_x_ and H_y_) modulate the effective magnetic permeability (i.e., μ_eff_, Figure S3) of the soft-magnetic thin film, thereby affecting the impedance of the graphene coil. As shown in Figure 3c, the planar graphene micro-coils are sandwiched between the soft magnetic thin films in an orthogonal manner, and the alternating magnetic field excited by them in the soft magnetic thin films can be accurately and quantitatively described by Maxwell’s equations [34]. Based on this, the mutual inductance and self-inductance of the *i*-th graphene wire can be expressed in the following forms, respectively (for details, see Supplementary Material S2):

(6) $$L_{s}^{i}=\frac{1}{Iw_{c}}\int_{-0.5w_{c}}^{0.5w_{c}}\Phi_{2}(x)dx=\frac{\mu_{0}\mu_{eff}t_{m}l_{c}}{2w_{c}}(1-2\frac{\lambda }{w_{c}}\frac{1+\xi -2e^{-\frac{w_{c}}{\lambda }}+(1-\xi )e^{-\frac{{2w}_{c}}{\lambda }}}{{\left(1+\xi \right)}^{2}-{\left(1-\xi \right)}^{2}e^{-\frac{{2w}_{c}}{\lambda }}})$$

(7) $$M_{i,j}=\frac{1}{Iw_{c}}\int_{d}^{d+w_{c}}\Phi_{3}\left(x\right)dx=\frac{\lambda }{Iw_{c}}\left(A_{3}e^{d/\lambda }\left(e^{w_{c}/\lambda }-1\right)+B_{3}e^{-d/\lambda }\left(1-e^{{-w}_{c}/\lambda }\right)\right)$$

Here $L_{s}^{i}$ and *M_i,j_* represents the self-inductance (i.e., the *i*-th one) and mutual inductance (i.e., between the *i*-th and *j*-th ones) of graphene respectively; Φ represents the magnetic flux generated around the graphene wire (i.e., the i-th one); $\lambda =\sqrt{t_{m}\mu_{eff}g/2}$, $\xi =tanh((l_{m}-w_{c})/2\lambda )$, $\alpha =\frac{l_{c}t_{m}{\mu_{0}\mu }_{eff}}{2}$; t_m_ (27 μm) represents the thickness of the soft magnetic thin strip, and *l*_m_ (45 mm) represents its length; while w_c_ (1 mm) and l_c_ (10 mm) correspond to the width and length of the graphene wire, respectively; d (1 mm) represents the distance between two adjacent graphene wires. In law with Greenhouse law [35], The inductance of the zigzag structure of graphene can be expressed as:

(8) $$L_{1}=L_{2}=L_{x}=L_{y}=\sum_{i=1}^{2N}L_{s}^{i}+\sum_{i=1,j\neq i}^{2N}q_{i,j}M_{i,j}$$

where N = 5 denotes the number of turns of zigzag structure, *q_ij_* takes values of 1 and −1 when the current directions (of the two wires) are identical and opposite, respectively.

### 3.2. The Sensitivity Performance of Graphene Flexible Magnetic Sensor

In order to achieve accurate detection of the 3D magnetic field, this paper conducts a detailed analysis of the electrical output characteristics of graphene flexible magnetic sensors under the action of magnetic fields in different directions. For the obtained experimental data, the MI ratio and MR ratio, respectively, represent the response capabilities of the sensor to magnetic field changes in the in-plane (i.e., x and y directions) and out-of-plane (i.e., z direction).

(9) $$\Delta Z_{Hx}/Z_{Hx}=\left[Z_{Hx}\left(H\right)-Z_{Hx}\left(H_{max}\right)\right]/Z_{Hx}\left(H_{max}\right)$$

(10) $$\Delta Z_{Hy}/Z_{Hy}=\left[Z_{Hy}\left(H\right)-Z_{Hy}\left(H_{max}\right)\right]/Z_{Hy}\left(H_{max}\right)$$

(11) $$\Delta R_{Hz}/R_{Hz}=\left[R_{Hz}\left(H\right)-R_{Hz}\left(H=0\right)\right]/R_{Hz}\left(H=0\right)$$

Here, *Z* and *R*(*H*) represent the impedance and resistance under the external dc magnetic field H, respectively. Z(H_max_) is the impedance corresponding to the maximum magnetic field H_max_.

**Figure 4:**
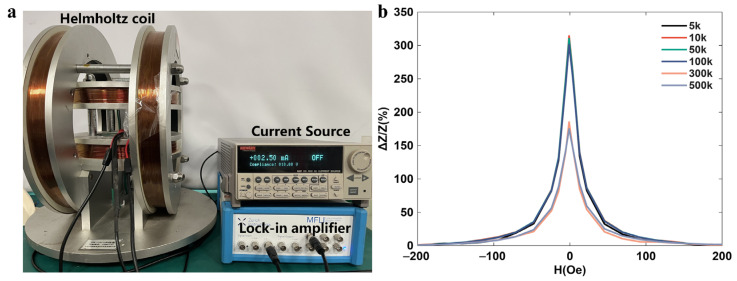
(**a**) Sensor magnetic sensitivity performance measurement system; (**b**) The changed impedance with the H_dc_ at different excitation frequencies.

Given that the sensor exhibits distinct MI performance under different excitation frequencies, this study first characterized the MI properties of the 3D graphene magnetic sensor under varying current excitation frequencies. The configuration of the measurement system is illustrated in Figure 4a. In the experiment, a Helmholtz coil was employed to generate a uniform dc magnetic field ranging from −200 to 200 Oe, a signal generator (Tektronix AFG3102C, Shanghai, China) supplied an alternating current with a peak value of 2.25 mA to the pads 2 and 7, and a lock-in amplifier (Zurich Instruments MLFI 5 M, Zurich, Switzerland) was employed to measure the output voltage *V_Hy_* of the sensor.

The experimental results show that when the in-plane dc magnetic field H_dc_ varies between −200 and 200 Oe and an alternating current of 10 kHz is applied through the excitation coil, the magneto-impedance ratio of the graphene-based magnetic sensor measured at pads 6 and 8 can reach the maximum value of 323% at the excitation frequency of 10 kHz. When the excitation frequency is lower than 10 kHz, the magneto-impedance ratio increases significantly with the increased frequency, indicating that the change of magneto-reactance caused by the magnetic induction effect between the excitation coil and sensing coil improves with the increased frequency. When the frequency exceeds 10 kHz, the magnetic domain wall relaxation effect leads to a decrease in the sensor’s response ability to the changing magnetic field. Additionally, the experiment also indicates that the magnetic permeability of FeSiB ribbon reaches a saturated state when the magnetic field strength is 110 Oe. Therefore, in order to ensure the optimal in-plane magnetic field sensing performance, the maximum magnetic field H_max_, and the frequency and peak value of excitation current are modified to 110 Oe, 10 kHz, and 2.25 mA in the subsequent tests, respectively.

Subsequently, this study systematically investigated the response characteristics of the sensor under the condition of a 3D magnetic field. All subsequent experiments were carried out in a magnetic shielding room, and by shielding the interference of the background magnetic field, the accuracy of the measurement results was effectively ensured. Specifically, the magnetic sensor located at the center of the Helmholtz coil was rotated by a high-precision rotating platform to comprehensively evaluate its directional sensitivity under various magnetic field directions.

#### 3.2.1. Response of the Sensor to H_z_

In this study, we conducted the impedance (i.e., *R_Hz_*, *Z_HX_*, and *Z_HY_*) tests of a 3D magnetic sensor to characterize its response characteristics to the 3D magnetic field.

**Figure 5:**
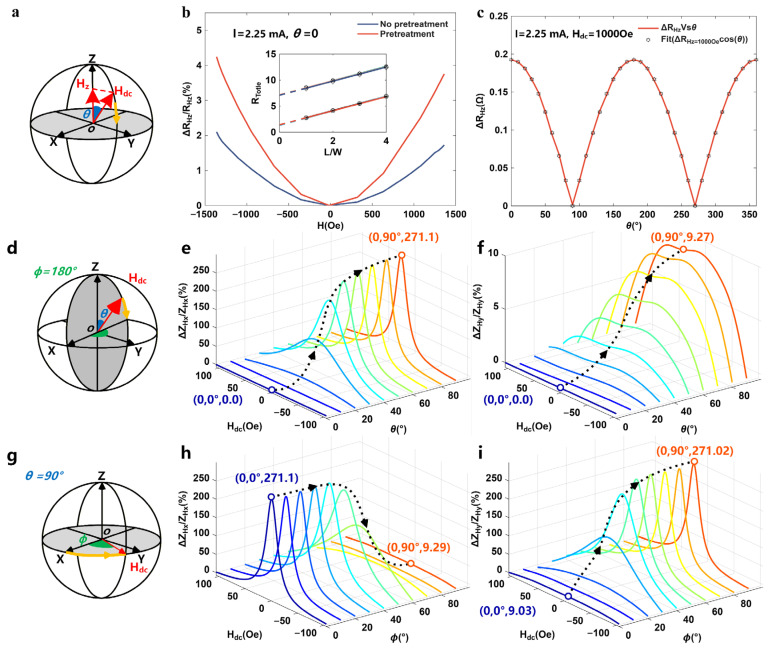
(**a**) Schematic diagram of out-of-plane MR effect test (i.e., R_Hz_); (**b**) Variation of R_Hz_ with H_dc_ in graphene (θ = 0°); (**c**) Magnetic field response of R_Hz_ with polar angle θ; (**d**) Schematic diagram of out-of-plane MI effect test; (**e**) Magnetic field response of Z_Hx_ with polar angle θ; (**f**) Magnetic field response of Z_Hy_ with polar angle θ; (**g**) Schematic diagram of in-plane MI effect test; (**h**) Magnetic field response of Z_Hx_ with azimuth angle φ; (**i**) Magnetic field response of Z_Hy_ with azimuth angle φ.

By collecting the output signal between sensor pads 4 and 5, the magnetoresistance performance of graphene material was analyzed by comparing that of graphene with and without IPr pretreatment (see Figure 5b). Here, the total resistance measured between pad 4 and pad 5 satisfies the formula R_tot_ = 2 × R_c_ + R_Gra_, where R_c_ represents the contact resistance at the interface between graphene and metal; R_Gra_ is the resistance of graphene itself. It can be seen in Figure 5b that the contact resistance R_c_ of the magnetic sensor with IPr pre-treatment decreases from 3.6 Ω to 0.7 Ω, which improves the maximum MR change rate from 2% to 4.3%. This is because the IPr pre-treatment can generate functional groups on the gold surface, which increases the number of conductive channels [31].

Figure 5c shows the variation trend of magnetoresistance when a dc magnetic field with a fixed amplitude of 1000 Oe rotates in the XOZ plane with the polar angle θ. The experimental results show that when a 1000 Oe dc magnetic field is applied in a direction perpendicular to the device surface (i.e., θ = 0°), Δ*R_Hz_* is initially positive; as the polar angle θ rotates to 90°, Δ*R_Hz_* shows a monotonically decreasing trend and finally drops to zero; then Δ*R_Hz_* gradually recovers when the polar angle θ continues to increase up to 180°. By fitting the measured data, it can be observed that there is a positive correlation between the magnetoresistance change (i.e., Δ*R_Hz_*) of the device and the absolute cosine value of polar angle θ (i.e., Δ*R_Hz_* ∝ |cos*θ*|). It can thus be concluded that Δ*R_Hz_* arising from the MR effect correlates exclusively with the *H_z_*.

When the dc magnetic field rotates with the polar angle θ, Figure 5d–f show the variation rules of Z_Hx_ and Z_Hy_ measured at pads 1–3 and pads 6–8 through the MI effect. In the experiment, when the polar angle θ is scanned from 0° to 90° in the XOZ plane, the maximum values of Δ*Z_Hx_*/*Z_Hx_* and Δ*Z_Hy_*/*Z_Hy_* both increase gradually from the initial zero value to the peak value. This behavior is because the demagnetization factor in the out-of-plane direction of the thin ribbon is much larger compared to that in the plane, the effective magnetic field in this direction approaches zero, and produces a negligible MI effect response. It can thus be concluded that Δ*Z_Hx_* and Δ*Z_Hy_* arising from the MI effect correlate exclusively with the *H_plane_* (i.e., *H_x_* or *H_y_*).

#### 3.2.2. Response of the Sensor to H_x_ and H_y_

Figure 5g–i show the variation trends of Δ*Z_Hx_*/*Z_Hx_* and Δ*Z_Hy_*/*Z_Hy_* measured at pads 1–3 and 6–8 when the dc magnetic field rotates with the azimuth angle φ. When φ is increased from 0° (i.e., the x-axis direction) to 90° (i.e., the y-axis direction), the maximum value of Δ*Z_Hx_*/*Z_Hx_* decreases from 271.1% to 9.29%, while the maximum value of Δ*Z_Hy_*/*Z_Hy_* increases sharply from 9.03% to 271.02%. This trend confirms that *Z_Hx_* and *Z_Hy_* exhibit the distinct directional selectivity to magnetic fields along the x- and y-axes, respectively.

The weak demagnetization factor of the magnetic thin film along its length direction (easy axis) enhances the sensitivity of *Z_Hx_* and *Z_Hy_* to magnetic field components aligned with their respective easy axes. Specifically, the sensitivities along the X and Y axes reach 5.98 mV/Oe (S_xx_) and 5.93 mV/Oe (S_yy_), respectively. In contrast, the magnetic field components perpendicular to the easy axis induce trivial impedance changes in *Z_Hx_* and *Z_Hy_*, with sensitivities of only 0.39 mV/Oe (S_xy_) and 0.37 mV/Oe (S_yx_), respectively. This magnetic field coupling effect can be effectively eliminated by synchronously collecting the *Z_Hx_* and *Z_Hy_* signals at pads 1–3 and 6–8. Specifically, the magneto-impedance variations ΔZ_Hx_ and ΔZ_Hy_ measured in the experiment can be expressed as:

(12) $${∆Z}_{Hx}=-S_{xy}H_{y}-S_{xx}H_{x}$$

(13) $${∆Z}_{Hy}=-S_{yy}H_{y}-S_{yx}H_{x}$$

After solving the above equations, the two components H_x_ and H_y_ of in-plane magnetic field can be expressed as:

(14) $$H_{x}=\frac{S_{xy}{∆Z}_{Hy}-S_{yy}{∆Z}_{Hx}}{S_{yy}S_{xx}-S_{xy}S_{yx}}$$

(15) $$H_{y}=\frac{S_{xx}{∆Z}_{Hy}-S_{yx}{∆Z}_{Hx}}{S_{xy}S_{yx}-S_{yy}S_{xx}}$$

The MI effect and MR effect exhibit symmetry with respect to the polarity of in-plane and out-of-plane magnetic fields, respectively. Therefore, the measured Z_Hx_, Z_Hy_, and R_Hz_ can only reflect the amplitudes of the magnetic field components in each direction (i.e., H_x_, H_y_, and H_z_), but cannot distinguish their polarities. Thus, the azimuth angle ϕ is limited to the range of 0° to 90° in the above measurement process. To provide the magnetic field polarity discrimination ability and simultaneously improve the detection sensitivity of the magnetic sensor near the magnetic field to be measured, this study introduces a permanent magnet to provide a bias magnetic field H_b_. Specifically, the direction of the bias magnetic field is set as ϕ = 45° and θ = 30°, whose intensity is H_b_ = 100 Oe. In this way, the original symmetry is broken, thereby achieving the effective discrimination of the external magnetic field’s polarity.

In addition, the bias magnetic field can tune the operating range of the magnetic sensor to the vicinity of each magnetic field component of H_b_, which provides the maximum sensitivity and significantly improves the overall performance of the sensor.

#### 3.2.3. Experimental Demonstration of 3D Magnetic-Field Sensing

**Figure 6:**
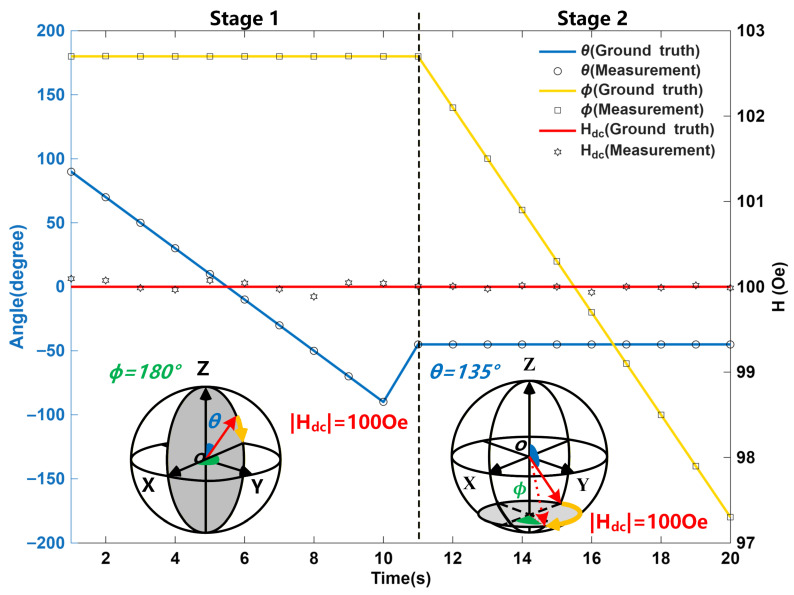
Measurement of the 3D magnetic field and corresponding ground truth.

To confirm the aforementioned decoupling approach and corresponding performance of the proposed planar graphene magnetic sensor for the 3D magnetic field detection, a dc magnetic field with the fixed strength of 100 Oe and variable spatial orientation was introduced in the experimental setup (Figure 6). Through quantifying the discrepancies between the measured amplitude/direction components of the magnetic field and the corresponding reference values, the measurement precision of the magnetic sensor was evaluated.

In this research, a sequential rotation technique including two steps was employed to conduct 3D magnetic field measurements. The experimental workflow is as follows: first, by keeping the azimuth angle constant, the polar angle is modified; then, by fixing the polar angle, the azimuth angle is rotated.

Stage 1: The external magnetic field was constrained to rotate within the XOZ plane, where the azimuth angle *ϕ* was fixed at 180° and θ (i.e., the polar angle) decreased step-by-step from 90° to −90° with a step value of 30°. During this stage, the detected magnetic field (i.e., *H*_x_, *H*_y_, *H*_z_) was converted to the corresponding components (i.e., $\theta ,\;\phi \;and\;H_{dc}$) in the spherical coordinate system in order to characterize the sensor’s magnetic field response in this plane.

Stage 2: The polar angle *θ* was held constant at 135°, while the azimuth angle ϕ of the external magnetic field was adjusted to sweep from 180° to −180°. This phase aimed to further validate the sensor’s directional sensitivity and stability to the magnetic fields with different orientations.

The experimental results show that the average relative error of the proposed flexible graphene magnetic sensor in 3D magnetic field measurement is only 0.036%, which indicates the high detection accuracy of magnetic fields in arbitrary directions. Notably, the sensor eliminates the need for multiple excitations to complete detection tasks, offering a key advantage in detection efficiency. The decoupling can be finalized in approximately 15 ns with a low-power microprogrammed control unit (MCU), realizing the detection of the time-varying 3-D magnetic field.

### 3.3. Equivalent Magnetic Noise

The detection limit of a magnetic sensor is fundamentally constrained by its equivalent magnetic noise, a relationship that can be mathematically described as:

(16) $$B_{in}=N_{V}/S_{A}$$

Here, *N_v_* (noise voltage) and *S_A_* (sensitivity) are defined as the sensor’s key performance metrics. Under an operating current of 2.25 mA, the sensitivities of the sensor in the three directions inside the magnetic shielding chamber are 59.8 V/T (H_x_), 59.3 V/T (H_y_), and 0.035 V/T (H_z_), respectively.

**Figure 7:**
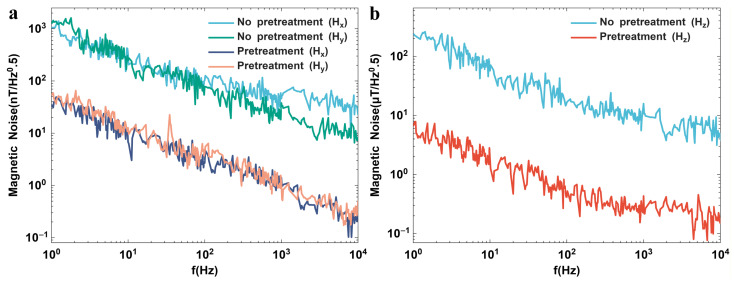
Equivalent magnetic noises of graphene flexible magnetic sensors in (**a**) in-plane and (**b**) out-of-plane directions.

Noise voltage characterization was conducted via a Keysight E4727A Advanced Noise Analyzer (Santa Rosa, CA, USA), with directional equivalent magnetic noises visualized in Figure 7. On one hand, the noise voltage of in-plane MI effects is mainly determined by 1/f magnetic noise dominated at the low-frequency range and the magnetization fluctuations of soft magnetic film at the high-frequency range. On the other hand, the model is formulated as:

(17) $$N_{V}=\frac{V_{0}^{\gamma }}{f^{\vartheta }}\frac{\alpha_{H}}{J}$$

The noise of MR effect mainly comes from 1/f flicker noise, and its characteristics conform to the Hooge noise model [36,37]. In Formula (12), V_0_ represents the voltage applied across the sample, $\alpha_{H}$ is the Hooge constant, and it has a positive correlation with the contact resistance $R_{c}^{2}$ (i.e., Figure 5c) [37].

(18) $$\alpha_{H}\propto R_{c}^{2}\times f(\mu )\times h\left(T\right)$$

where *f*(μ) = (1/μ)^δ^, the value range of parameter *δ* is approximately between 1.5 and 3 for the bent devices, μ is the carrier mobility; *h*(*T*) is related to the temperature-dependent trapping-detrapping process, density fluctuations, etc. Additionally, the exponents γ and ϑ take the values of 2 and 1, respectively, while J represents the total number of charge carriers.

The experimental results show that by pre-treating the metal surface with IPr to reduce the contact resistance R_c_ from 3.6 Ω to 0.7 Ω (i.e., Figure 5b), in the unbent state (natural state), after fitting with the experimental data, the Hooge constant $\alpha_{H}$ is reduced from 0.28 without pre-treatment to 0.011, and the equivalent magnetic noise of the magnetic sensor is reduced by about one order of magnitude (i.e., Figure 7). In the x, y and z directions, the equivalent magnetic noise of the designed magnetic sensor measures 31 nT/Hz^1/2^, 36 nT/Hz^1/2^ and 6992 nT/Hz^1/2^ at 1 Hz, respectively. In addition, by introducing a magnetic convergence structure, the sensitivity of the sensor in the z-direction can be effectively improved, thereby further enhancing the resolution in this direction (for details, see Supplementary Material S3).

We prepared a total of five devices, and the success rate reached 100%. Additionally, the measurement results show that all five devices exhibit the maximum MI ratio varying between 320.1% and 327% at the excitation current frequency of 10 kHz for the varied in-plane magnetic field, while the change of MR remains stable between 3.93% and 4% for the varied out-of-plane magnetic field. The experimental data show fair consistency, verifying the reliability of the preparation process. Next, we verified the long-term stability and anti-bending performance of the device.

### 3.4. Long-Term Stability and Bending Performance

**Figure 8:**
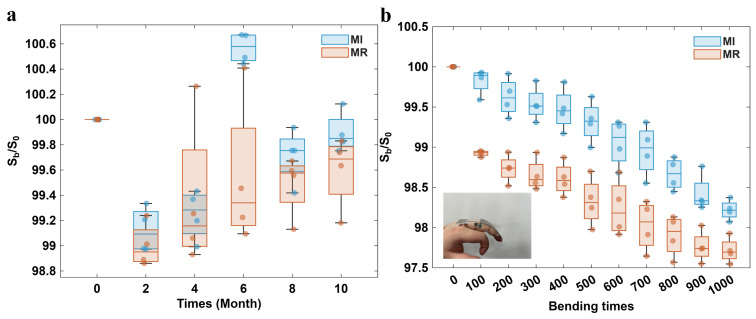
Test results of (**a**) long-term stability and (**b**) bending resistance characteristics of sensors.

We conducted long-term stability tests on the four fabricated devices. Specifically, these fabricated devices were stored in air at a constant room temperature, and their performances were measured every two months. The performances of the devices after different periods were characterized by the normalized magnetic field sensitivity, and the results are shown in Figure 8. After 10 months of observation, the magnetic field sensitivity of magnetic sensors remained at around 99.8% of the initial performance, and no aging phenomena, such as graphene-metal contact failure, were observed. This excellent long-term stability guarantees the practical application of magnetic sensors.

In addition, we have systematically evaluated the bending resistance performance of the magnetic sensor. The test method is as follows: the magnetic sensor was attached to the human finger joint with large deformations, and a total of 1000 bending operations were applied to the sensor. After a certain number of bending cycles, the changes of magnetic field sensitivity caused by the MI effect and the MR effect were measured, respectively, when the substrate returned to its natural state. Figure 8 shows the change trend of MR and MI sensitivity with increasing number of bending times. The experimental results show that even after 1000 bending cycles, the device can still maintain 97% of the initial sensitivity, demonstrating excellent bending resistance performance. These characteristics enable the magnetic sensor to have broad application prospects in the field of wearable electronic devices.

## 4. Discussion

The proposed planar 3D magnetic sensor based on a graphene microcoil sandwiched between PI/soft magnetic multilayer films utilizes the synergistic coupling of magneto-impedance (MI) and magnetoresistance effects to detect an external 3D magnetic field. This sensor only requires a planar microstructure processed by patterning and milliamp-level excitation current, which can complete the accurate and real-time 3D magnetic field detection with a single measurement.

The conventional 3D magnetic sensors are usually assembled with the 3D orthogonally arranged single-axis sensors (e.g., as shown in Table 2, GMR [21]/TMR/fluxgate/Hall [38]/AMR [13]), which results in severe three-axis alignment errors and a complex calibration procedure. Correspondingly, the planar 3D magnetic sensors have been reported to mitigate such issues. However, the reported planar 3D magnetic sensors require multiple excitations to measure the 3D magnetic field. For example, Li R. [39] developed a magnetic sensor based on the spin-orbit torque (SOT) effect. However, the sensor needs to apply a current density as high as 6.8 MA/cm^2^ to adjust the sensitive direction, which not only results in the extremely high-power consumption (140 mW) but also significantly increases the complexity of the sensing system.

Shiogai J. [40] proposed a planar device integrating the anomalous Hall effect (AHE), AMR, and unidirectional magnetoresistance (UMR) effects to sense the 3D magnetic field. However, this sensor requires multiple measurements and complex procedures to decouple the magnetic fields in the X, Y, and Z directions, which hinders the real-time measurement ability. More critically, neither of the above rigid plane 3D sensors possesses the flexible property, thus hindering their applications in smart wearable devices.

For comparison, the magnetic sensor proposed in this study effectively reduces the alignment errors between the three axes with the planar structure and improves the real-time measurement ability with only one time excitation. In addition, compared with the high-power consumption (usually in the mW range) of other three-dimensional sensors, the planar graphene three-dimensional magnetic sensor proposed in this study demonstrates significant low-power advantages (i.e., 76 μW). This characteristic makes it more competitive and practical in energy-consumption-sensitive application scenarios.

In conclusion, by benefiting from many advantages such as low power consumption, high sensitivity, excellent resolution, and outstanding anti-bending performance, this new type of 3D graphene sensor provides new solutions for energy-constrained application scenarios, such as smart wearable and health monitoring devices with the 3D high-precision magnetic sensing ability.

## Figures and Tables

**Table 2:** Performance comparison of 3-D magnetic sensors.

Sensor	Device Quantity	Geometry	Measurement Times	Flexible	Power	Resolution	Reference
AHE + UMR + AMR	1	2D	2	No	32 μW	N/A	[40]
SOT	1	2D	2	No	140.3 mW	1090 nT/Hz^1/2^ 1191 nT/Hz^1/2^ 657.6 nT/Hz^1/2^	[39]
SDT	1	2D	1	No	100 mW	1 nT/Hz^1/2^	[20]
GMR + fluxguide	4	3D	1	No	1.5~9.5 mW	3~9 nT/Hz^1/2^	[21]
Hall sensor	6	3D	1	No	N/A	N/A	[38]
AMR	3	3D	1	Yes	N/A	N/A	[13]
MI + MR	1	2D	1	Yes	76 μW	31 nT/Hz^1/2^ 36 nT/Hz^1/2^ 6992 nT/Hz^1/2^	This work

## Data Availability

The raw data supporting the conclusions of this article will be made available by the authors on request.

## Supplementary Materials

The following supporting information can be downloaded at: https://www.mdpi.com/article/10.3390/polym18040477/s1, S1: Measurement of mutual inductance coefficient k; S2: Inductor with the zigzag graphene sandwiched between the soft magnetic films; S3: Magnetic convergence structure; Figure S1: Measurement circuit of mutual inductance coefficient k. Figure S2: Schematic diagram of the sensor structure; Figure S3: MH curve of FeSiB ribbon; Figure S4: Impedance changes of graphene-based magneto-impedance (MI) sensors under the magnetic field along the easy axis; Figure S5. Magnetic convergence test fixture; Figure S6. Air gap of magnetic convergence structure L_a_ Vs. Magnetic amplification factor G_f_.
