# Supplementary material for: Low-Power Three-Dimensional Graphene-Based Flexible Magnetic Sensor
Source: Polymers (Basel). 2026 Feb 13;18(4):477. doi: 10.3390/polym18040477 (PMC12943843; doi:10.3390/polym18040477)
Supplement: Supplementary file 1 [file polymers-18-00477-s001.zip › polymers-4145696-supplementary.docx]

Supporting Materials

Low-power three-dimensional graphene-based flexible magnetic sensor

Shiliang Zhao^1^, Yao Wang^1*^

^1^State Key Laboratory of Submarine Geoscience, School of Automation and Intelligent Sensing, Shanghai Jiao Tong University, Shanghai 200240, China

* Corresponding Author: Yao Wang, Email: [yaowang898@sjtu.edu.cn](mailto:yaowang898@sjtu.edu.cn)

**S1 Measurement of mutual inductance coefficient k**

The mutual inductance coefficient k is obtained through experimental measurement. The test circuit is shown in Figure 3 of response letter (i.e., FigureS1 of revised manuscript), During the measurement process, the inductor L_1_ of graphene is first connected in series with a resistor R. Subsequently, a voltage V with fixed magnitude and frequency of 10 kHz is applied across the two ends (i.e. Pad 1 and 3) of sensor, so that the excitation current I can be obtained as following:

$I=\frac{V_{R}}{R}=2.25 mA$ (S1)


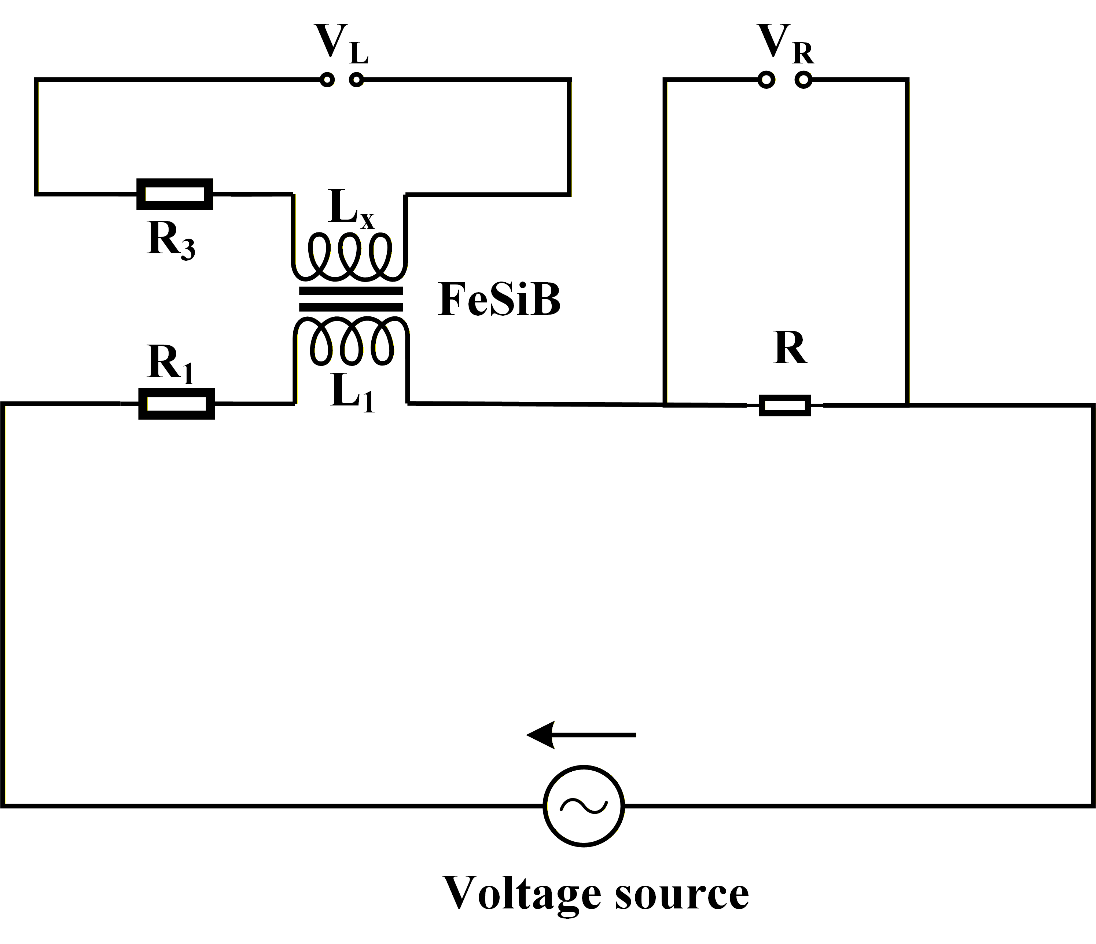


**Figure S1.** Measurement circuit of mutual inductance coefficient k.

According to the law of electromagnetic induction, the output voltage V_L_：

$V_{L}=M\frac{dI}{dt}=k\omega\sqrt{L_{1}L_{X}}I$ (S2)

In the formula, L_1_ = L_2_ = L_x_ = L_y_ = 650 μH, which are measured by the impedance analyzer. Therefore, the mutual inductance coefficient k can be obtained:

$k=\frac{V_{L}}{{\omega IL}_{1}}$ (S3)

Thus the value of parameter k is 0.989 according to the measurement results, which is approximated as 1 in the main text.

**S2 Inductor with the zigzag graphene sandwiched between the soft magnetic films**

The magnetic flux distribution of zigzag graphene interlayered between soft magnetic films is shown in **Figure S2c**. The alternating magnetic field from graphene's excitation current can be described using Maxwell's equations.

$\oint H\cdot dl=I$ (S4)

$\nabla\cdot B=0$ (S5)

For the structure in Fig S1c, the above equations can be expressed as:

$2H_{m}-\frac{\partial H_{g}}{\partial x}g=\frac{I}{l_{m}}$ (S6)

$H_{g}=t_{m}\mu_{eff}\frac{\partial H_{m}}{\partial x}$ (S7)

$\mu_{eff}=1+\frac{\mu_{r}-1}{1+N_{L}(\mu_{r}-1)}$ (S8)

where H_g_ denotes the magnetic field in the gap between the soft magnetic films, H_m_ represents the internal magnetic field within the soft magnetic films, g is the gap spacing between the upper and lower soft magnetic films, l_m_ and t_m_ are the length and thickness of the soft magnetic films, respectively, and $\mu_{eff}$ denotes the effective permeability of the soft magnetic films, $\mu_{r}$ is the relative permeability of the soft magnetic film, $N_{L}$is the demagnetization factor in the length direction of the soft magnetic film.


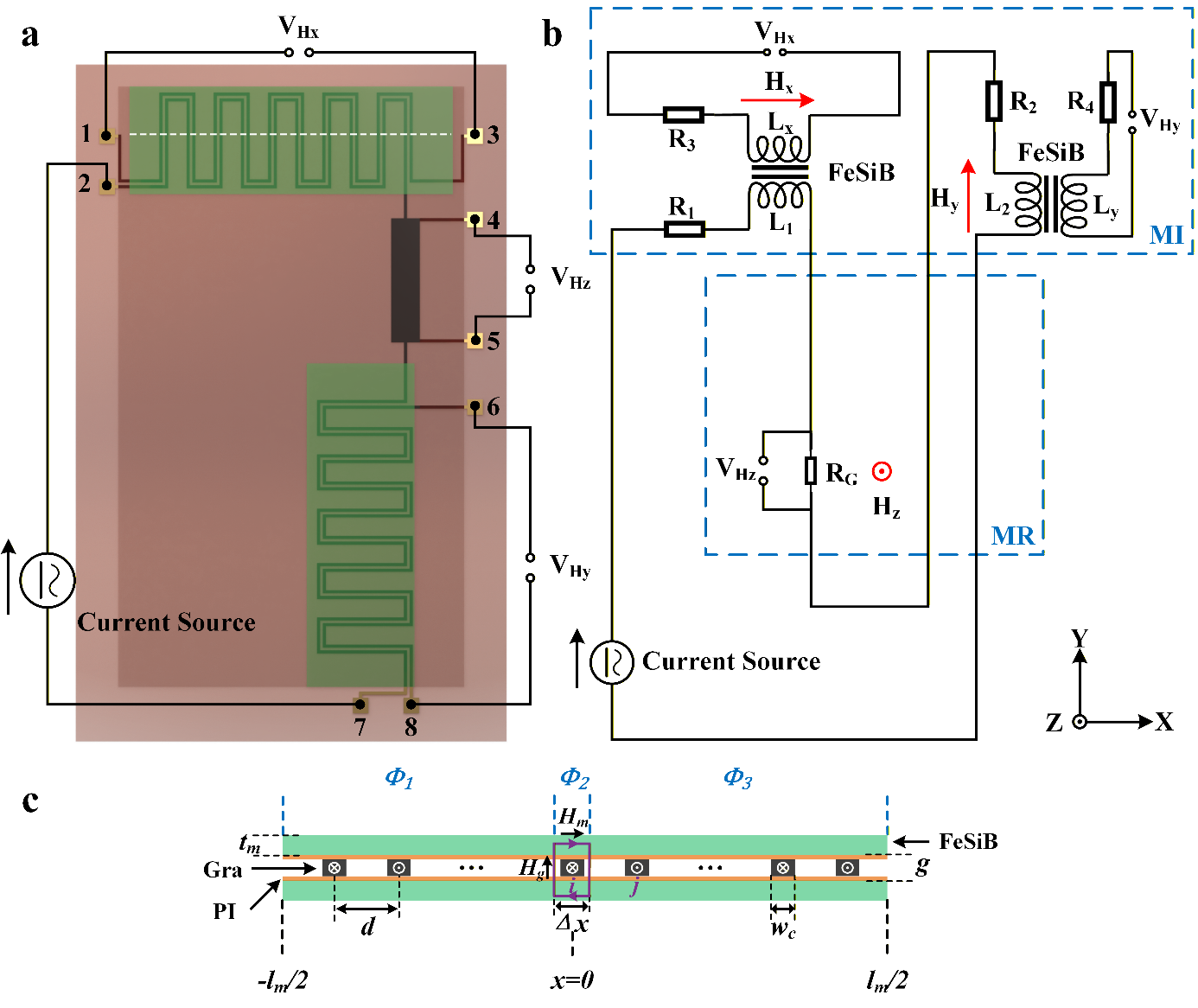


# Figure S2. Schematic diagram of the sensor structure. a magnetic sensor. b Equivalent circuit model. c Cross-sectional view of the device along the white dashed line of Figure S1a, and the magnetic flux distribution of the zigzag graphene line sandwiched between the soft magnetic layers is denoted as the yellow lines.

Combining the equation set S3 and S4:

$\frac{\partial^{2}\Phi}{\partial x^{2}}-\frac{\Phi}{\lambda^{2}}=-\frac{\theta}{\lambda^{2}}I$ (S9)

Where $\lambda=\sqrt{t_{m}\mu_{eff}g/2}$, *l*_m_ and *t*_m_ represent the length and thickness of the soft magnetic thin film respectively, while *l*_c_ and *w*_c_ represent the length and width of graphene line, respectively.

From equation S6, the magnetic flux of the graphene line and its adjacent region in Figure S1c can be expressed as:

$\Phi_{1}=A_{1}e^{\frac{x}{\lambda}}+B_{1}e^{-\frac{x}{\lambda}},x\in({-l_{m}}/2,{-w_{c}}/2)$ (S10a)

$\Phi_{2}=A_{2}e^{\frac{x}{\lambda}}+B_{2}e^{-\frac{x}{\lambda}}+\alpha I,x\in({-w_{c}}/2,{w_{c}}/2)$ (S10b)

$\Phi_{3}=A_{3}e^{\frac{x}{\lambda}}+B_{3}e^{-\frac{x}{\lambda}},x\in({-l_{m}}/2,{-l_{m}}/2)$ (S10c)

where $\alpha=\frac{l_{c}t_{m}{\mu_{0}\mu}_{eff}}{2}$, the boundary conditions are as follows:

$\Phi_{1}\left( -\frac{l_{m}}{2} \right)=\Phi_{3}\left( \frac{l_{m}}{2} \right)=0$ (S11a)

$\Phi_{1}\left( -\frac{w_{c}}{2} \right)=\Phi_{2}\left( -\frac{w_{c}}{2} \right),\Phi_{2}\left( \frac{w_{c}}{2} \right)=\Phi_{3}\left( \frac{w_{c}}{2} \right)$ (S11b)

$\frac{\partial\Phi_{1}\left( -\frac{w_{c}}{2} \right)}{\partial x}=\frac{\partial\Phi_{2}\left( -\frac{w_{c}}{2} \right)}{\partial x},\frac{\partial\Phi_{2}\left( \frac{w_{c}}{2} \right)}{\partial x}=\frac{\partial\Phi_{3}\left( \frac{w_{c}}{2} \right)}{\partial x}$ (S11c)

The six coefficients in equation S7 can be determined by solving the boundary conditions:

$A_{1}=-B_{1}*e^{\frac{l_{m}}{\lambda}}$ (S12a)

$B_{1}=\alpha I\frac{tanh\left( \frac{w_{c}}{2\lambda} \right)e^{\frac{w_{c}}{2\lambda}}}{\left( e^{\frac{w_{c}}{\lambda}}-e^{\frac{l_{m}}{\lambda}} \right)\tanh\left( \frac{w_{c}}{2\lambda} \right)-(e^{\frac{w_{c}}{\lambda}}+e^{\frac{l_{m}}{\lambda}})}$ (S12b)

$A_{2}=B_{2}=B_{1}*\frac{e^{\frac{w_{c}}{2\lambda}}+e^{\frac{l_{m}}{\lambda}}e^{-\frac{w_{c}}{2\lambda}}}{e^{\frac{w_{c}}{2\lambda}}-e^{-\frac{w_{c}}{2\lambda}}}$ (S12c)

$A_{3}=B_{1},B_{3}=A_{1}$ (S12d)

Therefore, the self-inductance and mutual inductance of graphene can be expressed as:

$L_{s}^{i}=\frac{1}{Iw_{c}}\int_{-0.5w_{c}}^{0.5w_{c}} \Phi_{2}(x)dx=\frac{\mu_{0}\mu_{eff}t_{m}l_{c}}{2w_{c}}(1-2\frac{\lambda}{w_{c}}\frac{1+\xi-2e^{-\frac{w_{c}}{\lambda}}+(1-\xi)e^{-\frac{{2w}_{c}}{\lambda}}}{\left( 1+\xi\right)^{2}-\left( 1-\xi\right)^{2}e^{-\frac{{2w}_{c}}{\lambda}}})$ (S10a)

$M_{i,j}=\frac{1}{Iw_{c}}\int_{d}^{d+w_{c}} \Phi_{3}\left( x \right)dx=\frac{\lambda}{Iw_{c}}\left( A_{3}e^{d/\lambda}\left( e^{{w_{c}}/\lambda}-1 \right)+B_{3}e^{{-d}/\lambda}\left( 1-e^{{{-w}_{c}}/\lambda} \right) \right)$ (S10b)

where $\xi=tanh((l_{m}-w_{c})/2\lambda)$.

According to the Greenhouse law, the inductance (L_1_ and L _2_) of graphene based excitation coil and inductance (L_x_ and L_y_) of sensing coil can be expressed as:

$L_{1}=L_{2}=L_{x}=L_{y}=\sum_{i=1}^{2N} L_{s}^{i}+\sum_{i=1,j\neq i}^{2N} q_{i,j}M_{i,j}$ (S11)

where N=5 is the turns of the zigzag structure, *M_i,j_* represents the mutual inductance between the *i*th and *j*th graphene wires, q_ij_=1 and −1 when the current direction is the same and opposite, respectively.

After substituting the measured permeability μ_eff_ change (i.e.Fig S3) of the FeSiB ribbon into Equation S11，Figure S4 presents the predicted impedance change of the graphene magneto-impedance (MI) sensor (gGra = 57μm) derived from the theoretical calculation results of Equation S11. The theoretical prediction curve exhibits strong consistency with experimental data, which validates the reliability of the theoretical model.


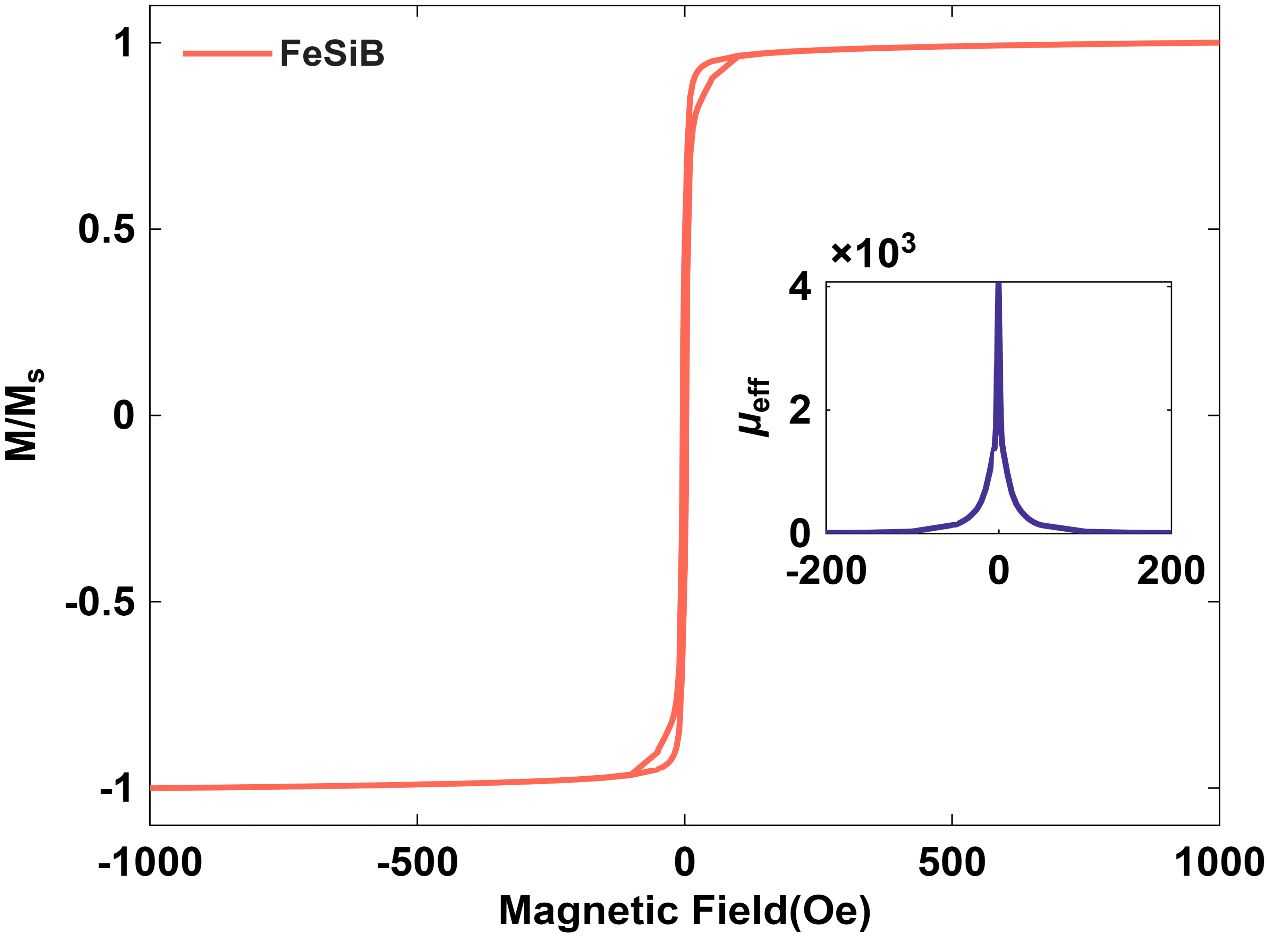


**Figure S3.** MH curve of FeSiB ribbon.


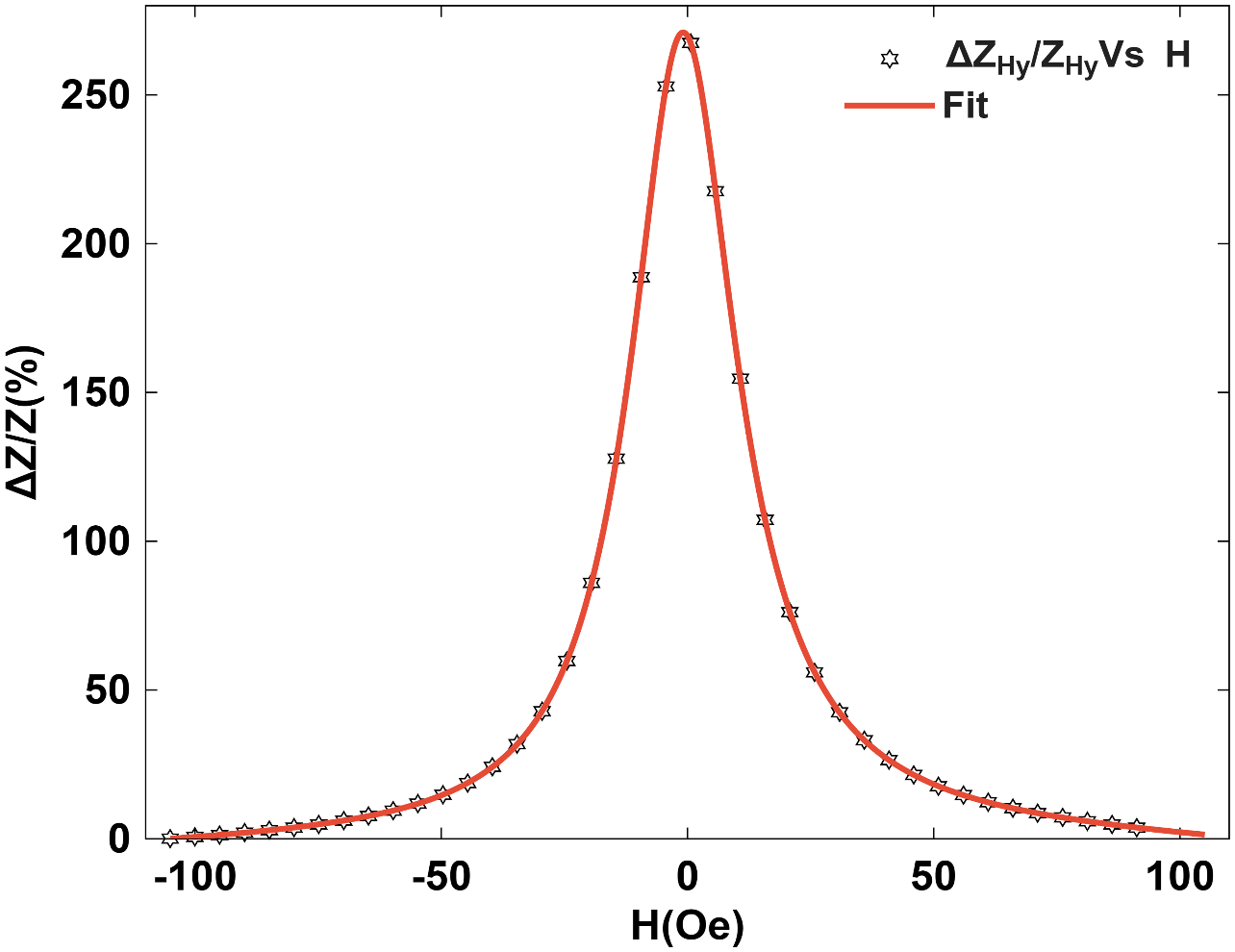


# Figure S4: Impedance changes of graphene-based magneto-impedance (MI) sensors under the magnetic field along the easy axis.

**S3 Magnetic convergence structure**

Currently, the magnetic field resolution of 6.99 μT in the out-of-plane direction can already meet the application scenarios such as gesture recognitions. Additionally, the magnetic field sensitivity and resolution along the out-of-plane direction (i.e., z direction) of magnetic sensor can be improved by concentrating the external magnetic field flux with the magnetic concentrator (magnification factor of G_f_), as shown in Figure 4 of response letter (i.e., Fig.S5 of revised manuscript). The material of magnetic concentrator is ferrite with a high relative magnetic permeability of about 5000, which introduce negligible magnetic field noise. And the shape of magnetic concentrator is “nail - shaped”, which can be installed on the designed test fixture. The air gap L_a_ between both tips of magnetic concentrators can be adjusted through the displacement platforms, which can adjust the sensitivity of sensor. For the gap L_a_ = 1mm, G_f_ = 120, the magnetic field resolution in the z - direction can be improved to 60 nT/Hz^1/2^, which can alleviate the imbalance of in - plane and out - of - plane resolution.


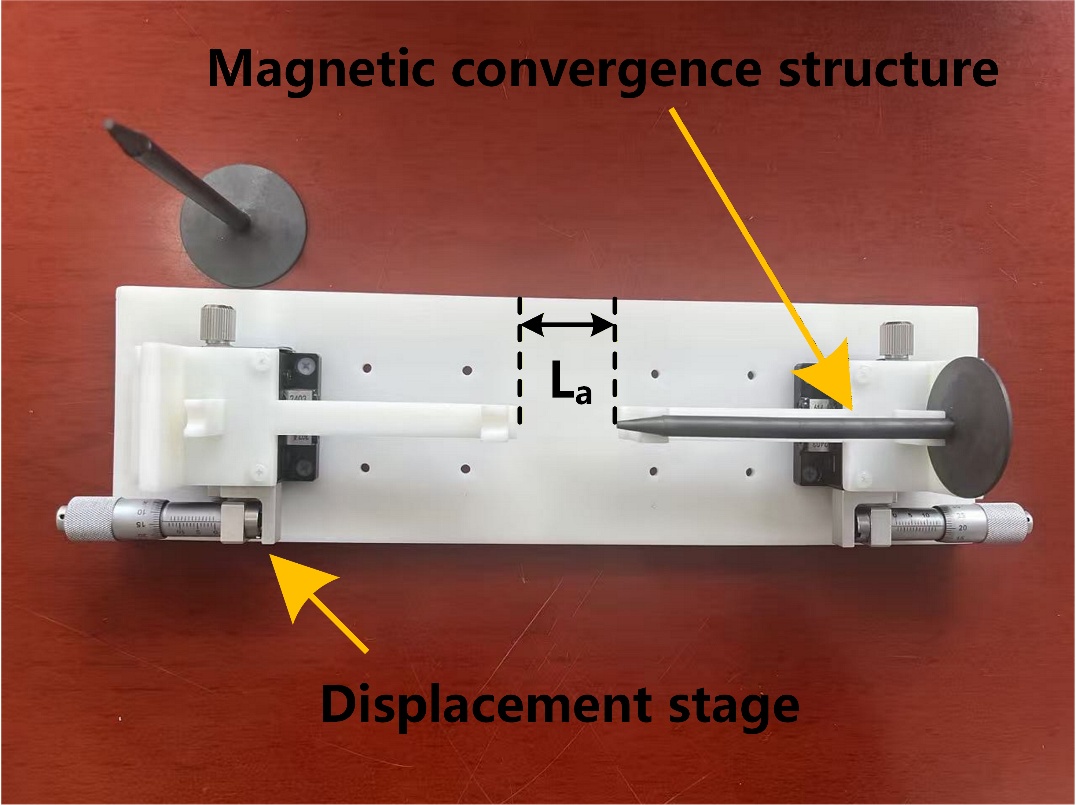


**Figure S5.** The magnetic concentrator. Improving the magnetic field sensitivity and resolution along the out-of-plane direction


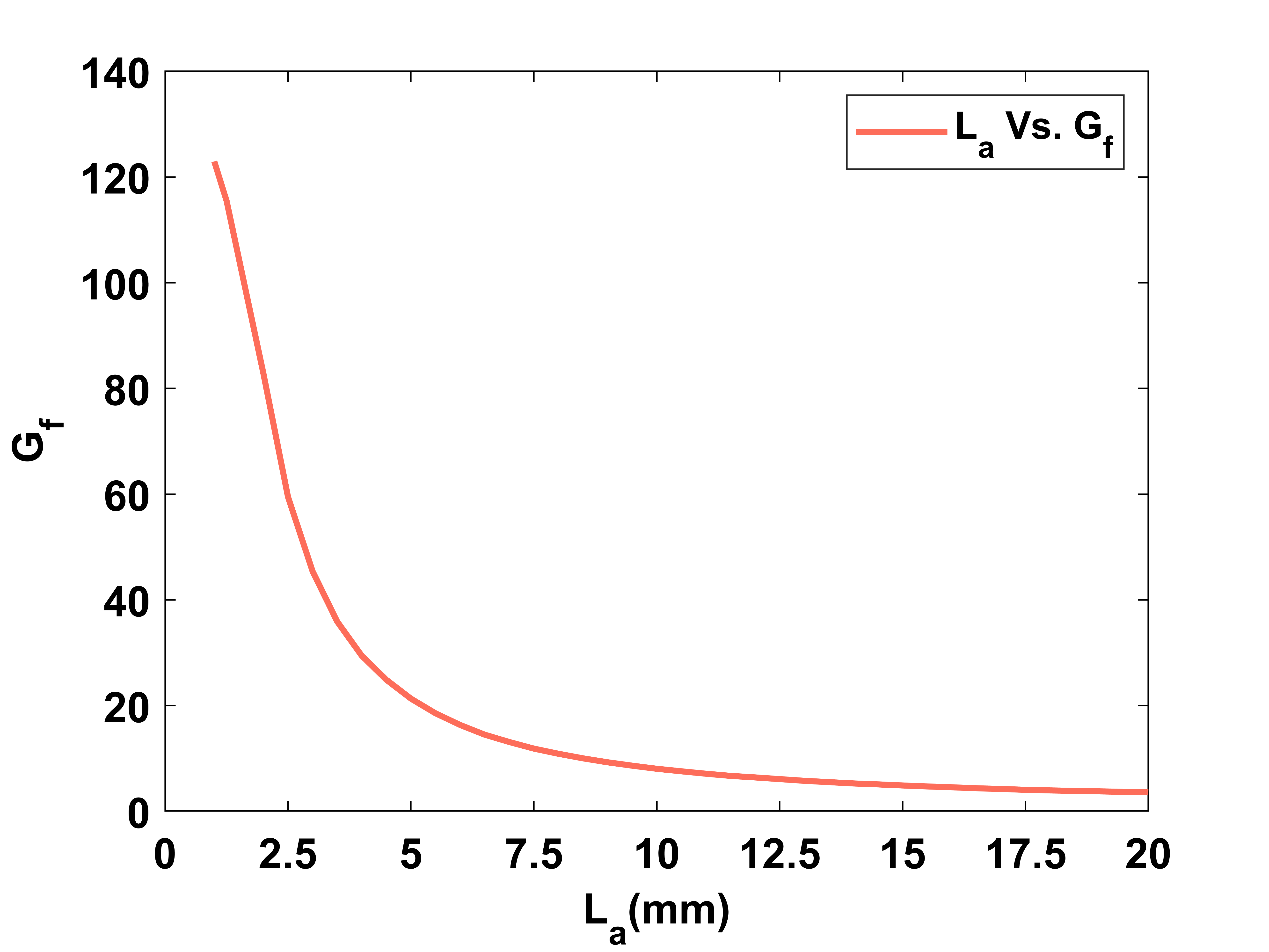


**Figure S6.** Magnetic amplification factor G_f_  as function of air gap La

\
